# The Optimal First‐Line Therapy for Extensive‐Stage Small‐Cell Lung Cancer Based on Liver Metastasis Status: A Network Meta‐Analysis and Systematic Review

**DOI:** 10.1002/cam4.70256

**Published:** 2024-10-02

**Authors:** Shu‐Ling Zhang, Jing Yu, Yuan Tian, Jie‐Hui Zhang, Li Sun, Le‐Tian Huang, Jie‐Tao Ma, Cheng‐Bo Han

**Affiliations:** ^1^ Department of Oncology Shengjing Hospital of China Medical University Shenyang China; ^2^ Innovative Cancer Drug Research and Development Engineering Center of Liaoning Province Shenyang China; ^3^ Department of Immunology, School of Basic Medical Science China Medical University Shenyang China

**Keywords:** immunotherapy, liver metastases, meta‐analysis, small‐cell lung cancer

## Abstract

**Purpose:**

To compare the efficacy of first‐line regimens based on programmed cell death (or ligand) [PD‐(L)1] blockade in extensive‐stage small‐cell lung cancer (ES‐SCLC) patients with or without liver metastases (LM), and to identify optimal treatment strategies.

**Methods:**

Network meta‐analysis of randomized controlled trials (RCTs) comparing chemo‐immunotherapy (CIT) and chemotherapy (CT) in ES‐SCLC patients stratified by LM. Overall survival (OS) and progression‐free survival (PFS) were evaluated using hazard ratios (HRs) and 95% confidence intervals (CIs).

**Results:**

Seven RCTs involving 3658 ES‐SCLC patients (1243 with LM, 2415 without LM) were analyzed. For patients with LM, the combination therapies of anti‐PD‐1 + CT (HR, 0.67; 95% CI, 0.54%–0.82%; *p* < 0.001) and anti‐PD‐L1 + CT + anti‐angiogenesis (HR, 0.84; 95% CI, 0.71%–0.99%; *p* = 0.042) demonstrated superior efficacy in prolonging OS compared to CT alone. The anti‐PD‐1 + CT regimen had the highest cumulative probability of 91.6% for extending OS in patients with LM. For patients without LM, all CIT regimens resulted in improved OS compared to CT alone, with the regimen of anti‐angiogenesis + anti‐PD‐L1 + CT ranking first and having the highest cumulative probability of 95.5% for prolonging OS.

**Conclusions:**

CIT is effective for ES‐SCLC patients regardless of LM status. For patients with LM, PD‐1 blockade combined with CT is the best option. For patients without LM, the most beneficial regimen is the combination of anti‐angiogenesis, PD‐L1 blockade, and CT.

## Graphical abstract

Chemo‐immunotherapy is effective for SCLC patients with liver metastases (LM). PD‐1 blockade combined with chemotherapy (CT) is optimal for LM patients. Non‐LM patients benefit most from anti‐angiogenesis, PD‐L1 blockade, and CT.

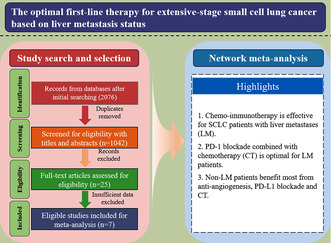

## 1 Introduction

Small‐cell lung cancer (SCLC), characterized by a high proliferative rate, a strong propensity for early metastasis, and a poor prognosis, accounts for approximately 15% of all lung cancers [1]. Nearly 75% of SCLC patients present with extensive‐stage (ES) disease with distant metastases at diagnosis [2]. The liver is one of the most common sites of metastasis, occurring in approximately 25% of patients with ES‐SCLC [3]. Over the past two decades, platinum‐based chemotherapy (CT) with etoposide has been the standard of care for ES‐SCLC [4], achieving a median overall survival (OS) of only approximately 10 months [5].

In recent years, immune checkpoint inhibitors (ICIs), including programmed death 1 (PD‐1) and programmed death ligand 1 (PD‐L1) inhibitors, have shown remarkable progress in improving the prognosis of patients with ES‐SCLC by restoring T‐lymphocyte function in antitumor immunity. The landmark IMpower133 [6] and CASPIAN [7] studies indicated that first‐line anti‐PD‐L1 atezolizumab or durvalumab in combination with CT significantly prolonged OS in patients with ES‐SCLC compared to CT alone. Based on these studies, combination therapies with atezolizumab or durvalumab have become the standard first‐line treatments for patients with ES‐SCLC. Subsequent studies have demonstrated a significant survival benefit with new combination regimens, including CT + anti‐PD‐1 (serplulimab [8], tislelizumab [9], toripalimab [10]), CT + anti‐PD‐L1 in combination with anti‐cytotoxic T‐cell lymphocyte antigen 4 (CTLA‐4), or anti‐angiogenic agents (anlotinib) [11].

A recent study [12] proposed that the tumor microenvironment (TME) in liver metastases (LM) of lung cancer exhibits low immune activation, resulting in a poor organ‐specific tumor response to immunotherapy. Results from the CASPIAN trial [7] showed that durvalumab in combination with CT prolonged OS in patients without LM, but not in patients with LM. However, the ASTRUM‐005 trial [8] indicated that serplulimab in combination with CT prolonged OS compared to CT alone in patients with ES‐SCLC with or without LM. Therefore, the importance of adding ICIs to CT in patients with LM‐SCLC requires further validation. We conducted this meta‐analysis with a large sample size to assess the value of various first‐line chemo‐immunotherapy (CIT) regimens in patients with LM‐SCLC.

## 2 Methods

### 2.1 Literature Search

According to the Preferred Reporting Items for Systematic Reviews and Meta‐Analyses (PRISMA) 2020 guidelines, we performed a systematic literature search of PubMed, Embase, Web of Science, Cochrane Library, and ClinicalTrials.gov databases for articles published in English from inception to June 10, 2024. The following search terms were used: (small cell lung cancer NOT non‐small cell) AND (immunotherapy OR immune checkpoint inhibitor OR CTLA‐4 OR PD‐1 OR PD‐L1) AND (clinical trial). In addition, references in all retrieved review articles, primary trials, and abstracts from the World Conference on Lung Cancer, American Society of Clinical Oncology, European Society for Medical Oncology, and American Association for Cancer Research meetings were screened using a computerized search, supplemented by manual searches.

### 2.2 Study Selection

Studies were included if they met the following criteria: (1) randomized controlled trials (RCTs); (2) studies in treatment‐naïve patients; (3) studies comparing survival outcomes (progression‐free survival [PFS] and/or OS) in patients with ES‐SCLC treated with first‐line CTLA‐4/PD‐1/PD‐L1 inhibitors (monotherapy or combination strategies) versus CT; (4) studies reporting hazard ratios (HRs) for survival analysis (PFS or OS) or numbers of events for relevant clinical endpoints for subgroups stratified by LM status; and (5) studies reporting original data enabling calculation of HRs or *p* values. Studies were excluded if they met one of the following criteria: (1) incomplete data reporting; (2) case reports, letters, editorials, meta‐analyses, systematic reviews, commentaries, duplicates, updated data in subsequent reports, single‐arm trials, observational studies, or nonclinical studies; (3) survival data that could not be extracted or converted from the original research; and (4) small sample sizes (*n* < 10). Two investigators (S.‐L.Z. and J.Y.) independently performed the study search and selection by examining titles and abstracts to identify potentially relevant studies. Disagreements were resolved through consensus with a third investigator (J.‐T.M.).

### 2.3 Data Extraction

Two investigators (S.‐L.Z. and J.Y.) independently screened information from eligible trials and recorded the following data in electronic spreadsheets: trial name, trial phase, title, year of publication or conference presentation, first author, number of participants, stage, treatment regimen, sample size evaluable for LM status, and PFS and/or OS according to LM status.

### 2.4 Quality Assessment

Two reviewers (L.S. and J.‐H.Z.) independently assessed the quality of RCTs using the Cochrane risk of bias (RoB) 2 tool [13]. Disagreements between the investigators were resolved by consensus.

### 2.5 Statistical Analysis

A meta‐analysis was performed to estimate the pooled HRs and 95% confidence intervals (CIs) for PFS and OS in patients with ES‐SCLC. Heterogeneity was assessed using *I* ^2^ values. If *I* ^2^ statistic > 50% or *p* < 0.05 indicated significant heterogeneity among the included studies, a random‐effects model was used for analysis. Otherwise, a fixed‐effects model was used. Publication bias was detected using funnel plots, and Begg's and Egger's tests. Leave‐one‐out sensitivity analysis was then performed. All reported *p*‐values were two‐tailed, and a *p*‐value below 0.05 was considered statistically significant.

R language (R version 4.3.2) was used for the network meta‐analysis. The ranking of treatment effects was assessed using rank probability plots, forest plots, league tables, and the area under the cumulative ranking curve. In the assessment of the efficacy of PFS and OS indices, an HR < 1.0 was considered to indicate that the treatment was more beneficial to the patient than the other treatments. In indirect comparisons, a CI crossing 1.0 was considered to indicate no statistical significance.

## 3 Results

### 3.1 Literature Review

As shown in Figure S1, after the evaluation of 2076 studies, seven phase III RCTs [6, 7, 8, 9, 14, 15, 16] that met the criteria were finally included in the meta‐analysis. These trials included 3658 patients with ES‐SCLC: 1243 with LM and 2415 without LM. Patients in these RCTs received five first‐line treatment strategies: CT alone, anti‐PD‐1 + CT, anti‐PD‐L1 + CT, anti‐PD‐L1 + anti‐CTLA‐4 + CT, and anti‐PD‐L1 + anti‐angiogenesis + CT. The detailed characteristics of each study are presented in Table 1.

**TABLE 1:** Characteristics of the included randomized controlled trials in this network meta‐analysis.

Trials (year)	Phase	LM (yes/no)	Regimens	Regimen type	HRs for OS of LM vs. non‐LM (95% CI)
ASTRUM‐005 (2022)	III	150/435	EC + serplulimab vs. EC + placebo	CT + anti‐PD‐1 vs. CT	0.58 (0.40, 0.84) vs. 0.62 (0.48, 0.80)
CAPSTONE‐1 (2022)	III	147/315	EC + adebrelimab vs. EC + placebo	CT + anti‐PD‐L1 vs. CT	0.92 (0.65, 1.31) vs. 0.61 (0.46, 0.81)
IMpower133 (2021)	III	149/254	EC + atezolizumab vs. EC + placebo	CT + anti‐PD‐L1 vs. CT	0.75 (0.52, 1.07) vs. 0.76 (0.56, 1.01)
KEYNOTE‐604 (2020)	III	187/266	EP + pembrolizumab vs. EP + placebo	CT + anti‐PD‐1 vs. CT	0.75 (0.55, 1.02) vs. 0.82 (0.62, 1.08)
RATIONALE‐312 (2023)	III	123/334	EC/EP + tirelizumab vs. EC/EP + placebo	CT + anti‐PD‐1 vs. CT	0.65 (0.44, 0.99) vs. 0.75 (0.59, 0.97)
ETER701 (2024)	III	158/335	EC + benmelstobart + anluotinib vs. EC + placebo	CT + anti‐PD‐L1 + anti‐angiogenesis vs. CT	0.79 (0.53, 1.18) vs. 0.51 (0.36, 0.72)
CASPIAN (2019)	III	221/316	EC/EP + durvalumab + tremelimumab vs. EC/EP + placebo	CT + Anti‐PD‐L1+ Anti‐CTLA‐4 vs. CT	0.90 (0.68, 1.20) vs. 0.74 (0.58, 0.96)
		212/325	EC/EP + durvalumab vs. EC/EP + placebo	Anti‐PD‐L1 + CT vs. CT	0.87 (0.66, 1.16) vs. 0.68 (0.53, 0.88)

Abbreviations: CI, confidence interval; CT, chemotherapy; CTLA‐4, cytotoxic T‐cell lymphocyte antigen 4; EC, carboplatin + etoposide; EP, cisplatin + etoposide; HRs, hazard ratios; LM, liver metastases; OS, overall survival; PD‐1, programmed death 1; PD‐L1, programmed death ligand 1; PFS, progression‐free survival.

### 3.2 Direct Analysis of PFS and OS Benefits With First‐Line CIT According to LM Status

**FIGURE 1:**
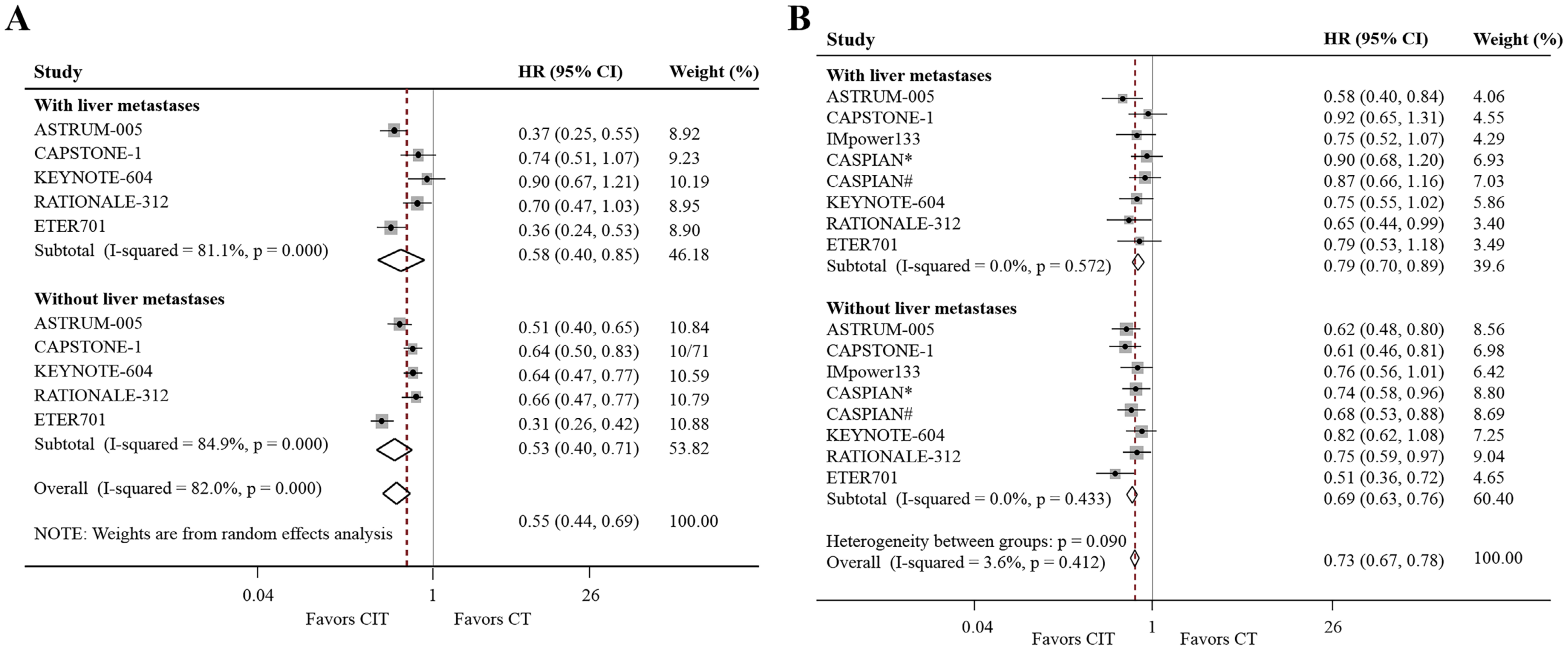
Effects of CIT on PFS and OS, according to LM status. Forest plot of PFS (A) and OS (B) comparing CIT with CT alone in SCLC patients with or without LM. CIT, chemo‐immunotherapy; CT, chemotherapy; LM, liver metastases; OS, overall survival; PFS, progression‐free survival; SCLC, small‐cell lung cancer. *Durvalumab plus tremelimumab plus chemotherapy group. ^#^Durvalumab plus chemotherapy group.

Compared with CT, CIT‐based treatment significantly prolonged PFS (HR, 0.58; 95% CI, 0.40%–0.85%; *p* = 0.005; and HR, 0.53; 95% CI, 0.40%–0.71%; *p* < 0.001, respectively; Figure 1A) and OS (HR, 0.79; 95% CI, 0.70%–0.89%; and HR, 0.69; 95% CI, 0.63%–0.76%, respectively; *p*_all_ < 0.001; Figure 1B) in patients with ES‐SCLC with or without LM.

To further evaluate the effects of different interventions on survival in patients with ES‐SCLC, we performed subgroup analyses of PFS and OS in patients with ES‐SCLC treated with anti‐PD‐1 + CT versus CT and anti‐PD‐L1‐based treatment + CT versus CT.

**FIGURE 2:**
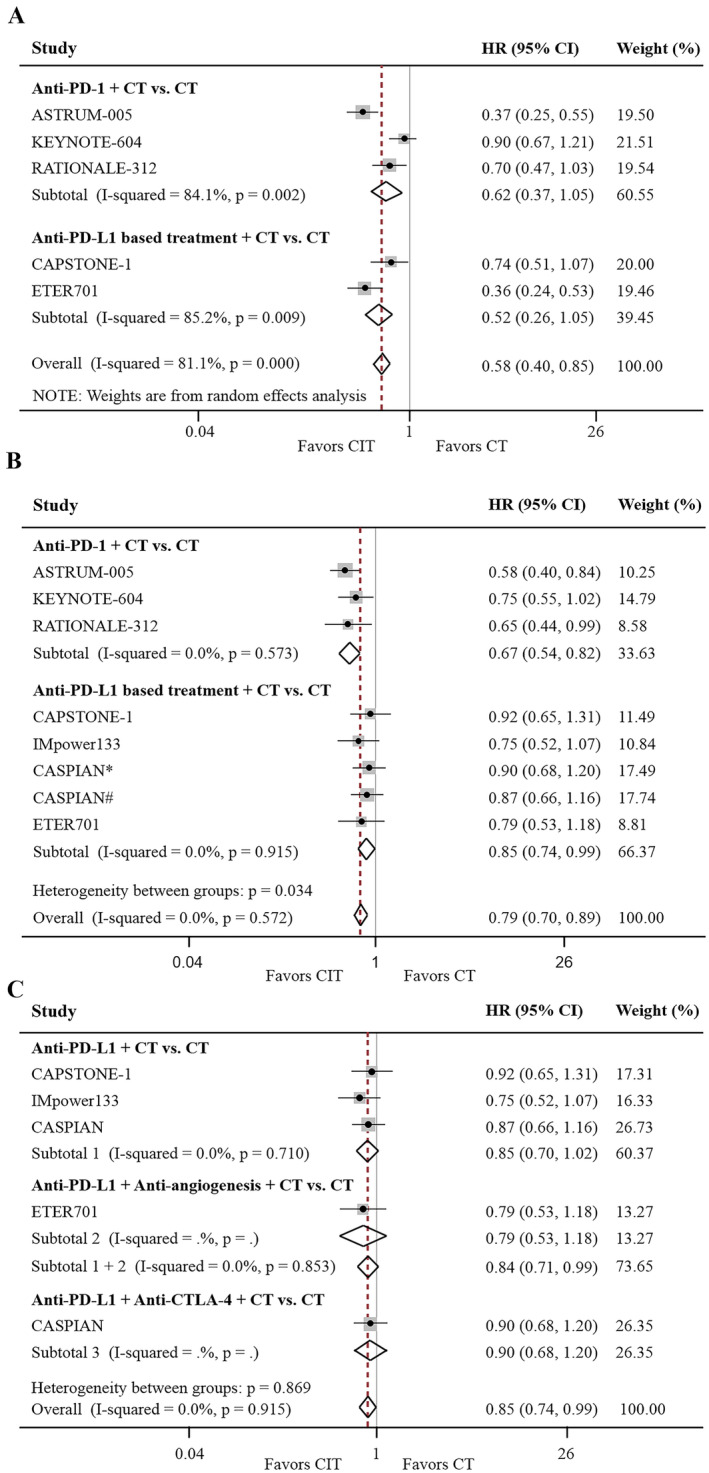
Effects of CIT on PFS and OS in SCLC patients with LM. Forest plot of PFS (A) and OS (B) comparing CIT with CT stratified by anti‐PD‐1 and anti‐PD‐L1 treatment. Forest plot of OS according to PD‐L1 blockade‐based treatment (C), including subgroups of anti‐PD‐L1 plus CT, CTLA‐4 plus anti‐PDL1 plus CT, and anti‐PD‐L1 plus anti‐angiogenesis plus CT. anti‐CTLA‐4, cytotoxic T‐cell lymphocyte antigen 4 inhibitor; anti‐PD‐(L)1, programmed cell death (ligand) 1 inhibitor; CIT, chemo‐immunotherapy; CT, chemotherapy; LM, liver metastases; OS, overall survival; PFS, progression‐free survival; SCLC, small‐cell lung cancer. *Durvalumab plus tremelimumab plus chemotherapy group. ^#^Durvalumab plus chemotherapy group.

Compared with CT alone, both anti‐PD‐1 + CT and anti‐PD‐L1‐based treatment + CT did not prolong PFS (HR, 0.62; 95% CI: 0.37%–1.05%; *p* = 0.074; and HR, 0.52; 95% CI, 0.26%–1.05%; *p* = 0.068, respectively; Figure 2A), but improved OS in SCLC patients with LM (HR for OS, 0.67; 95% CI, 0.54%–0.82%; *p* < 0.001; and HR, 0.85; 95% CI, 0.74%–0.99%; *p* = 0.034, respectively; Figure 2B). Although anti‐PD‐L1‐based therapy exhibited a trend towards a reduced risk of death in patients with LM compared with CT alone (HR, 0.86; 95% CI, 0.74%–1.01%; *p* = 0.068; and HR, 0.85; 95% CI, 0.70%–1.02%; *p* = 0.088; Figure S2 and Figure 2C, respectively), this was not significant when excluding the ETER701 trial or both the ETER701 and CASPIAN (anti‐PD‐L1 + anti‐CTLA‐4 + CT group) trials. However, excluding only the CASPIAN trial (anti‐PD‐L1 + anti‐CTLA‐4 + CT group) restored the significant benefit of anti‐PD‐L1 therapy over CT alone (HR, 0.84; 95% CI, 0.71%–0.99%; *p* = 0.042; Figure 2C), suggesting that the OS benefit may be due to the addition of anlotinib.

**FIGURE 3:**
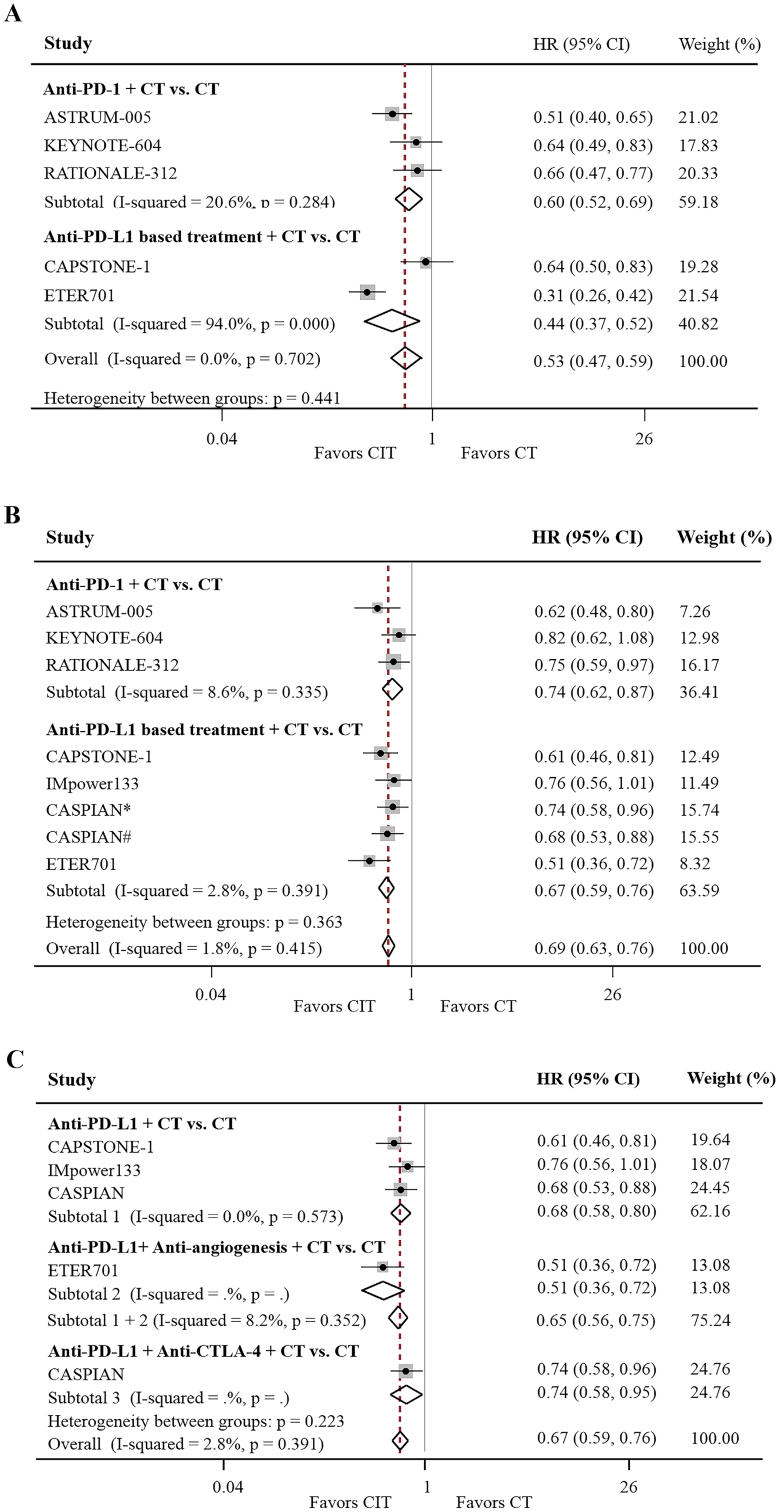
Effects of CIT on PFS and OS in SCLC patients without LM. Forest plot of PFS (A) and OS (B) comparing CIT with CT stratified by anti‐PD‐1 and anti‐PD‐L1 treatment. Forest plot of OS according to PD‐L1 blockade‐based treatment (C), including subgroups of anti‐PD‐L1 plus CT, CTLA‐4 plus anti‐PD‐L1 plus CT, and anti‐PD‐L1 plus anti‐angiogenesis plus CT compared with CT. anti‐CTLA‐4, cytotoxic T‐cell lymphocyte antigen 4 inhibitor; anti‐PD‐(L)1, programmed cell death (ligand) 1 inhibitor; CIT, chemo‐immunotherapy; CT, chemotherapy; LM, liver metastases; OS, overall survival; PFS, progression‐free survival; SCLC, small‐cell lung cancer. *Durvalumab plus tremelimumab plus chemotherapy group. ^#^Durvalumab plus chemotherapy group.

In patients with ES‐SCLC without LM, CIT was superior to CT alone in both the anti‐PD‐1 and anti‐PD‐L1 subgroups (HR for PFS, 0.53; 95% CI, 0.47%–0.59%; *p* < 0.001; HR for OS, 0.69; 95% CI, 0.63%–0.76%; *p* < 0.001; Figure 3A,B). Furthermore, the superiority of anti‐PD‐L1 + CT to CT alone in terms of OS improvement was sustained even when the CASPIAN and/or ETER701 trials were excluded (Figure 3C).

### 3.3 Network Meta‐Analysis of PFS and OS Benefit With First‐Line CIT According to LM Status

**FIGURE 4:**
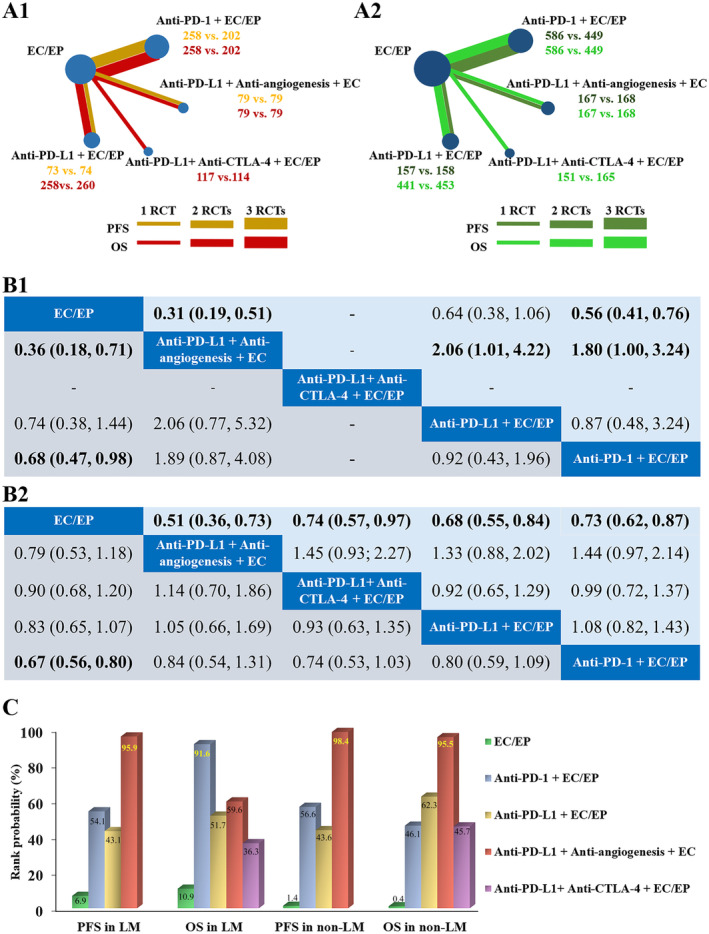
Network meta‐analysis results. Network plot of the included studies (A) node size is proportional to the number of included patients with LM (A1) and without LM (A2); Pooled estimate results (B) of HRs with 95% CIs in patients with LM (lower triangle) and without LM (upper triangle) for the comparison of column‐defining treatment versus row‐defining treatment: HRs with 95% CIs of PFS (B1) and OS (B2); ranking profiles of treatments for patients with SCLC (C). anti‐CTLA‐4, cytotoxic T‐cell lymphocyte antigen 4 inhibitor; anti‐PD‐1, programmed cell death 1 inhibitor; anti‐PD‐L1, programmed cell death ligand 1 inhibitor; CI, confidence interval; CIT, chemo‐immunotherapy; EC, etoposide plus carboplatin; EP, etoposide plus cisplatin; HR, hazard ratio; LM, liver metastases; OS, overall survival; PFS, progression‐free survival; RCTs, randomized controlled trials; SCLC, small‐cell lung cancer.

Figure 4A shows all strategies involved in the network meta‐analysis for PFS and OS in patients with and without LM.

Anti‐PD‐1 + CT was superior to CT alone in terms of PFS in patients with LM. No statistical differences were observed among the other CIT regimens, including the combination of anti‐CTLA‐4 + anti‐PD‐L1, anti‐angiogenesis + anti‐PD‐L1, and anti‐PD‐L1/anti‐PD‐1, with CT. In contrast, all CIT regimens were superior to CT alone in terms of PFS in patients without LM. The combination of anti‐angiogenesis + anti‐PD‐L1 + CT achieved a longer PFS than both anti‐PD‐1 + CT and anti‐PD‐L1 + CT (HR, 1.80; 95% CI, 1.00%–3.24%; and HR, 2.06; 95% CI, 1.01%–4.22%, respectively). No significant difference was observed between anti‐PD‐1 + CT and anti‐PD‐L1 + CT (HR, 0.87; 95% CI, 0.48%–2.24%) (Figure 4B1).

In terms of OS in patients with LM, no CIT regimens except anti‐PD‐1 + CT, demonstrated a significant prolongation of OS (HR, 0.67; 95% CI, 0.56%–0.80%), compared with CT alone. In contrast, for patients without LM, all types of CIT regimens achieved longer OS than CT alone. Nevertheless, the indirect comparison results revealed no significant differences between the various CIT regimens, regardless of the presence of LM (Figure 4B2).

### 3.4 Rank Probabilities

The Bayesian rank probabilities for all comparable treatments with respect to PFS and OS are shown in Figure 4C. Anti‐angiogenesis + anti‐PD‐L1 + CT had the highest probability of prolonging PFS regardless of the presence of LM (cumulative probabilities: 95.9% and 98.4%, respectively), and CT alone ranked last in terms of PFS (cumulative probabilities: 6.9% and 1.4%, respectively). For the ranking of OS outcomes in patients with LM‐SCLC, the order from best to worst performance was as follows: anti‐PD‐1 + CT, anti‐angiogenesis + anti‐PD‐L1 + CT, anti‐PD‐L1 + CT, anti‐CTLA‐4 + anti‐PD‐L1 + CT, and CT. In contrast, the rank order suggested that anti‐angiogenesis + anti‐PD‐L1 + CT was most effective in prolonging OS in patients without LM, with a cumulative probability of 95.5%. The treatments ranked from best to worst in terms of OS prolongation in patients with ES‐SCLC without LM were anti‐angiogenesis + anti‐PD‐L1 + CT, anti‐PD‐L1 + CT, anti‐PD‐1 + CT, anti‐CTLA‐4 + anti‐PD‐L1 + CT, and CT.

### 3.5 Study Quality and Sensitivity Analysis

The risk of bias in each study is detailed in Figure S3, and the overall quality of the trials was considered moderate to high.

Sensitivity analysis by sequential exclusion of each eligible study showed that the HR for SCLC patients with LM or without LM for each trial was comparable to the overall pooled HR (0.86, 95% CI, 0.83%–0.89%; and 0.82, 95% CI, 0.79%–0.84%; Figures S4 and S5).

### 3.6 Publication Bias

Funnel plots demonstrated no obvious publication bias existed (Figures S4 and S5). The *p*‐values for Begg's test and Egger's test across the seven RCTs for patients with LM were 0.834 and 0.904, and for patients without LM were 0.904 and 0.066, respectively.

## 4 Discussion

In this meta‐analysis, we analyzed updated data and compared various first‐line treatment regimens for patients with ES‐SCLC, with or without LM. Subgroup analysis involving direct comparisons revealed that compared to CT alone, anti‐PD‐1 + CT, but not anti‐PD‐L1 + CT, significantly prolonged OS in patients with SCLC with LM. The addition of anti‐angiogenesis therapy compensated for the lack of OS benefits of anti‐PD‐L1 + CT in patients with LM. In particular, indirect comparisons from the network analysis for survival further confirmed the findings of direct analysis and demonstrated that anti‐PD‐1 + CT was most likely to provide the longest OS, followed by anti‐PD‐L1 + anti‐angiogenesis + CT in patients with SCLC with LM. Anti‐angiogenesis + anti‐PD‐L1 + CT was found to be more suitable than other treatments for prolonging OS in patients with ES‐SCLC without LM.

Studies have shown that the combination of ICIs, such as anti‐PD‐1, anti‐PD‐L1, and anti‐CTLA‐4, in combination with CT improves survival compared to CT alone in patients with ES‐SCLC [17, 18]. However, previous studies have shown that the addition of ICIs to CT does not prolong OS or PFS in SCLC patients with LM [19, 20]. A recent meta‐analysis [21] indicated a potential trend towards improvement (*p* = 0.05), but only included RCTs until 2022 and therefore did not include survival outcomes from the ASTUM‐005, RATIONALE‐312, and ETER701 trials reported in 2023 and 2024. These high‐quality studies have further strengthened the evidence of the efficacy of adding ICIs to CT in SCLC patients with LM and complemented the lack of results in the above meta‐analysis. Additionally, this meta‐analysis is the first to explore the optimal treatment regimens by comparing different types of ICIs plus CT with CT alone in patients with LM. In addition to the direct analysis, we conducted a network meta‐analysis to indirectly compare different treatment strategies to determine the optimal choice for prolonging survival in patients with or without LM. Overall, this study provides valuable information and adds to the existing body of knowledge on this topic.

The findings of this study suggest that anti‐PD‐1 + CT may be more effective than anti‐PD‐L1 alone in prolonging OS in patients with SCLC and LM. Several possible reasons may explain this difference: first, anti‐PD‐1 antibodies can block PD‐1 on the surfaces of tumor‐infiltrating T cells, thereby simultaneously disrupting the binding of PD‐1 to both PD‐L1 and PD‐L2, leading to a more comprehensive inhibition of the immune escape pathway, whereas anti‐PD‐L1 antibodies only disrupt the PD‐1/PD‐L1 interaction, potentially allowing some cancer cells to evade immune surveillance via the PD‐1/PD‐L2 axis [22]; second, differences in the subclasses of IgG antibodies used in anti‐PD‐1 and anti‐PD‐L1 monoclonal drugs may lead to varying efficacies due to their distinct mechanisms of inhibiting T‐cell suppression, such as through the regulation of phosphatidylinositol‐3‐kinase and protein kinase B phosphorylation; for example, the fully humanized IgG4 monoclonal antibody serplulimab, which targets the PD‐1 receptor and covers a solvent‐accessible overlapping surface area of 445 Å^2^ (55% of the PD‐L1 surface), has demonstrated potent PD‐L1 and PD‐L2 blocking activity, increasing T‐cell responses and cytokine production in vitro [23]; third, the efficacy of immunotherapy usually depends on various tumor progression mechanisms and the complex TME [24]. Thus, the heterogeneity in the TME within liver metastases in individual patients may be a major factor explaining the slight differences between PD‐1 and PD‐L1 blockade treatments. Additionally, other factors, including unbalanced baseline characteristics and subsequent treatments, should also be considered. Therefore, the slight difference in OS between PD‐1 and PD‐L1 blockade treatments should be interpreted with caution, and further studies are needed to verify this.

The liver is generally considered an immune‐tolerant organ owing to its T‐cell anergy and immunosuppressive signals [25]. These characteristics can lead to the development of an immune desert, which may negatively affect the response to immunotherapy and lead to shorter survival in patients with LM [26, 27]. Studies have shown that vascular endothelial growth factor (VEGF) secreted by Kupffer cells and hepatic stellate cells in the liver has a negative immunoregulatory effect, whereas the addition of anti‐VEGF agents reverses the immunosuppressive TME by enhancing antigen presentation and T‐cell activation, and even increasing T‐cell infiltration into tumors after VEGF receptor (VEGFR) blockade [28]. The IMpower150 trial suggested that immunotherapy combined with anti‐angiogenic drugs is a potential treatment option for patients with advanced NSCLC with LM [29]. Similarly, for SCLC patients with LM, results from the ETER701 trial have shown that the addition of the anti‐angiogenic agent anlotinib to CIT leads to a longer PFS than that achieved with CT alone [16]. Notably, the present study also demonstrated the value of adding the antiangiogenic agent anlotinib to CIT as a first‐line treatment for these patients on the basis of the discernible OS prolongation after inclusion of the ETER701 trial (HR, 0.84; 95% CI, 0.71%–0.99%). Despite the lack of comparison between anti‐angiogenic agents plus CIT and CIT alone, our results suggest that anti‐angiogenesis treatment in combination with CIT could also be considered as an alternative treatment strategy for SCLC patients with LM.

In the highly immunosuppressive hepatic microenvironment, dysfunction and/or insufficient quantity of effector T cells are the main factors contributing to the unfavorable therapeutic efficacy of immunotherapy in patients with LM‐SCLC. Recent studies have revealed that the advantages of immunotherapy arise from the recruitment of fresh T cells from lymph nodes adjacent to the TME [30]. Therefore, attracting functional T cells to tumor areas in liver metastases is key to increasing therapeutic efficacy and must be further explored.

Our findings have substantial implications for both clinical and academic research. First, treatment with PD‐1 or PD‐L1 blockade is preferable to SCLC patients with LM, as the survival time exceeds that achieved with CT alone. Second, for SCLC patients with LM, therapeutic efficacy may be unfavorable due to dysfunction and insufficiency of effector T cells in the TME; therefore, treatments that improve the immunosuppressive microenvironment, such as VEGFR blockade, should be considered to enhance the response to immunotherapy by modulating the TME. Our network meta‐analysis indicated that the addition of anti‐angiogenic agents to CIT ranked first in prolonging PFS but not OS in patients with LM. Thus, in addition to anti‐angiogenesis treatment, other methods to improve the microenvironment or enhance antigen release should be considered, especially during the maintenance phase of first‐line anti‐angiogenesis therapy combined with immunotherapy. Third, although anti‐PD‐1 + CT showed a slightly longer OS than anti‐PD‐L1 + CT in patients with LM, this finding should be interpreted with caution, and future research should focus on improving the efficacy of both anti‐PD‐1 and anti‐PD‐L1 rather than determining the preferred treatment. Finally, the combination of anti‐PD‐L1 + anti‐CTLA‐4 + CT did not show advantages over other CIT strategies in improving survival, but still exhibited OS superiority over CT alone in ES‐SCLC patients without LM. Xie et al. [31] found that durvalumab + tremelimumab + CT provided OS benefits compared to durvalumab + CT in ES‐SCLC patients, particularly in those with high CD4 and MHC I and II gene signatures. This underscores the need for personalized medicine approaches to improve outcomes. However, given the higher incidence of adverse events with this regimen compared to PD‐(L)1 blockers [32], it should be carefully considered when treating ES‐SCLC patients.

This study has several limitations, including the heterogeneity of the included studies in terms of LM definition, other organ metastases, and individual‐level clinicopathological characteristics. This heterogeneity may have impacted the robustness of our findings and highlights the need for standardized criteria in future investigations. Toxicity is a critical component in the decision‐making process for treatment modalities. A recent meta‐analysis indicated that durvalumab + tremelimumab + CT presented a higher likelihood of causing adverse events grade ≥ 3 compared to other regimens when evaluating the safety of these combination therapies in patients with ES‐SCLC [17]. Although anti‐PD‐L1 + CT (22%–26%) and anti‐PD‐1 + CT (20%–31%) had similar probabilities of causing grade ≥ 3 adverse events, the addition of PD‐1/PD‐L1 inhibitors to chemotherapy tended to increase toxicity compared with chemotherapy alone, especially when a PD‐1 inhibitor was added [33]. Despite the lack of comprehensive data on toxicity profiles stratified by LM status, preventing a thorough evaluation of toxicity‐related outcomes, reported studies can provide useful references for the use of CIT in patients with LM in clinical practice.

## 5 Conclusion

This meta‐analysis was based on high‐quality RCTs and included the largest sample sizes to date. Despite the aforementioned limitations, this study provides valuable insights into the optimal treatment strategies for patients with SCLC with LM and highlights the importance of adding ICIs to CT to prolong survival outcomes. First‐line CIT, compared with CT alone, significantly prolonged PFS and OS in patients with ES‐SCLC, regardless of LM status. For patients with SCLC with LM, first‐line anti‐PD‐1 therapy appears to be more effective in prolonging survival than anti‐PD‐L1 therapy when combined with CT. However, the difference in OS between anti‐PD‐1 and anti‐PD‐L1 therapies was small. Our findings highlight the value of anti‐vascular treatment in patients with SCLC with LM treated with CIT, and the addition of antiangiogenic agents could enhance the efficacy of immunotherapy through VEGFR blockade. In contrast, in patients without LM, the combination of anti‐angiogenesis + anti‐PD‐L1 + CT or anti‐PD‐L1 + CT was preferable to anti‐PD‐1 + CT for prolonging OS, although all types of CIT regimens prolonged OS beyond that observed with CT alone.

## Author Contributions

**Shu‐Ling Zhang:** conceptualization (equal), data curation (equal), formal analysis (equal), funding acquisition (lead), investigation (equal), methodology (equal), writing – original draft (equal), writing – review and editing (equal). **Jing Yu:** data curation (equal), methodology (equal), writing – review and editing (equal). **Yuan Tian:** data curation (equal), resources (equal), writing – review and editing (equal). **Jie‐Hui Zhang:** data curation (equal), software (equal), writing – review and editing (equal). **Li Sun:** resources (equal), software (equal), validation (equal), writing – review and editing (equal). **Le‐Tian Huang:** investigation (equal), methodology (equal), validation (equal), writing – review and editing (equal). **Jie‐Tao Ma:** conceptualization (equal), investigation (equal), resources (equal), writing – original draft (equal), writing – review and editing (equal). **Cheng‐Bo Han:** conceptualization (equal), project administration (lead), resources (equal), visualization (lead), writing – review and editing (equal).

## Ethics Statement

The authors have nothing to report.

## Consent

The authors have nothing to report.

## Conflicts of Interest

The authors declare no conflicts of interest.

## Registration Information of Meta‐Analysis Protocol

The meta‐analysis protocol was registered in the International Platform of Registered Systematic Review and Meta‐analysis Protocols (registration no. INPLASY202460024).

## Supporting information


Data S1.

Data S2.

## Data Availability

The datasets used and/or analyzed during the current study are available from the corresponding author upon reasonable request.
