# Supplementary material for: The Optimal First‐Line Therapy for Extensive‐Stage Small‐Cell Lung Cancer Based on Liver Metastasis Status: A Network Meta‐Analysis and Systematic Review
Source: Cancer Med. 2024 Oct 2;13(18):e70256. doi: 10.1002/cam4.70256 (PMC11447196; doi:10.1002/cam4.70256)
Supplement: Supplementary file 1 — Data S1. [file CAM4-13-e70256-s002.pdf]

Figure S1. *Flowchart of selected randomized controlled trials included in this meta-analysis.*

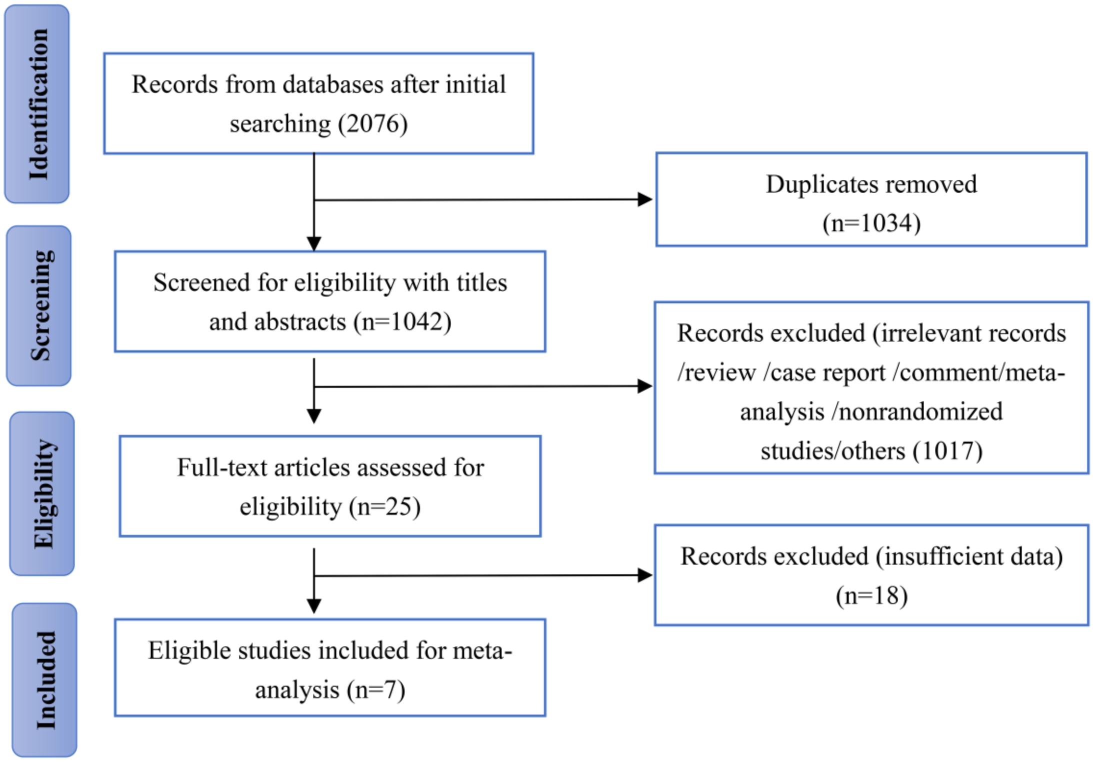

FigureS2. *Effects of CIT on OS in patients with LM*

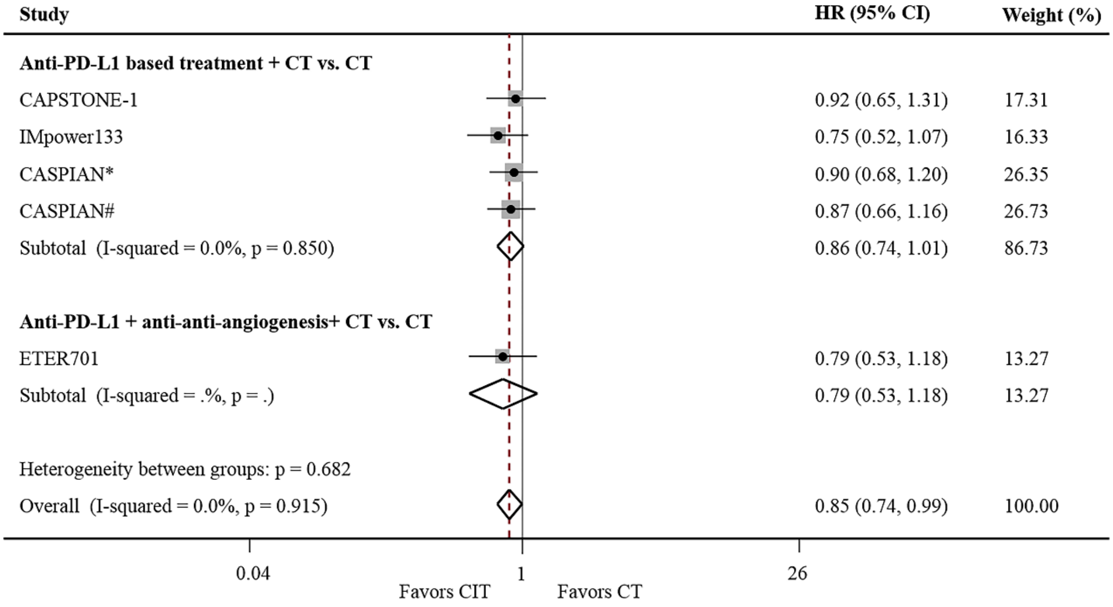

Notes: CIT, chemo-immunotherapy; CT, chemotherapy; OS, overall survival; SCLC, small cell lung cancer; PD-L1, programmed death ligand 1; LM, liver metastatic disease; *, durvalumab plus tremelimumab plus chemotherapy group; #, durvalumab plus chemotherapy group.

Figure S3. *Risk of bias graph: review authors' judgements about each risk of bias item presented as percentages across all included studies*

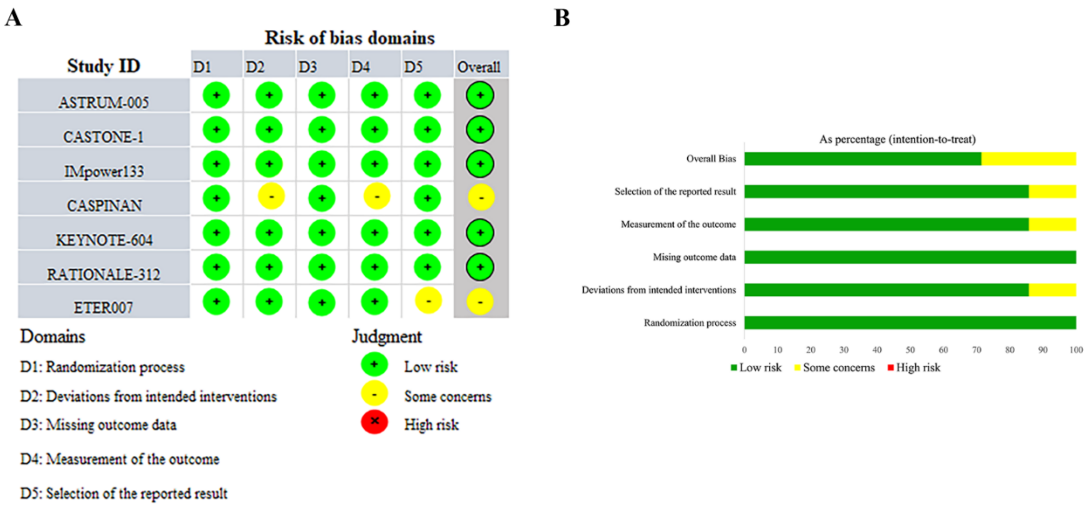

Figure S4. *Sensitivity analysis and publication bias results in patients with liver metastases.*

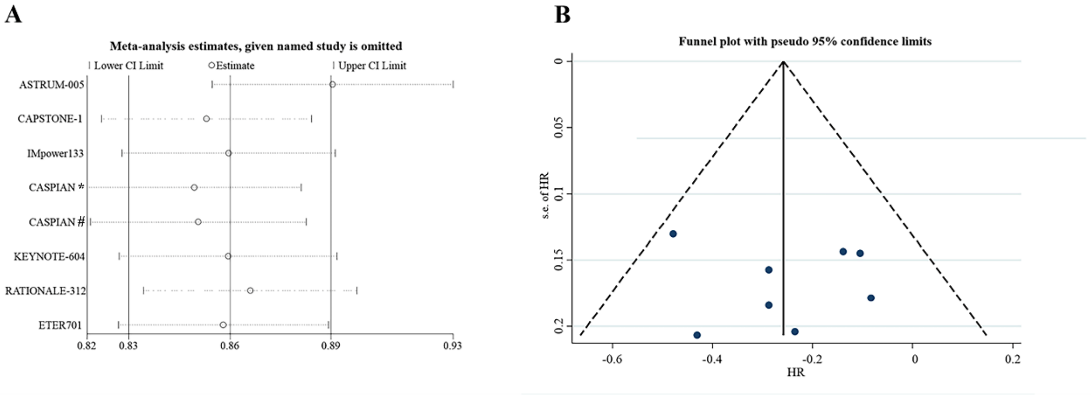

Notes: *, durvalumab plus tremelimumab plus chemotherapy group; #, durvalumab plus chemotherapy group.

Figure S5. *Sensitivity analysis and publication bias results in patients without liver metastases.*

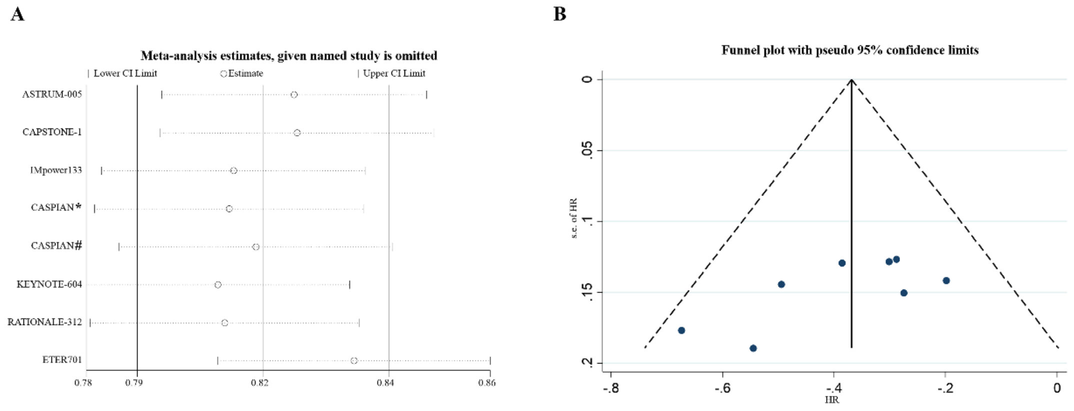

Notes: *, durvalumab plus tremelimumab plus chemotherapy group; #, durvalumab plus chemotherapy group.
