# Supplementary material for: The Optimal First‐Line Therapy for Extensive‐Stage Small‐Cell Lung Cancer Based on Liver Metastasis Status: A Network Meta‐Analysis and Systematic Review
Source: Cancer Med. 2024 Oct 2;13(18):e70256. doi: 10.1002/cam4.70256 (PMC11447196; doi:10.1002/cam4.70256)
Supplement: Supplementary file 2 — Data S2. [file CAM4-13-e70256-s001.docx]

Network meta-analysis is a generalisation of pairwise meta-analysis that compares all pairs of treatments within a number of treatments for the same condition. The graph-theoretical approach for network meta-analysis uses methods was equivalent to the frequentist approach to network meta-analysis which is based on weighted least squares regression.

#network analysis

The random effects model was used and HR was used as effect size.

netmeta <- netmeta(TE = lnHR,

seTE = selnHR,

treat1 = treat1,

treat2 = treat2,

studlab = study,

data = data_HR,

sm = "HR",

random = TRUE,

reference.group = "Control",

sep.trts = " vs ")

#graph plot

netgraph(m.netmeta,

points=T,

plastic=F,

col = "black",

col.points = "#BFBFBF",

bg.points = "#5C8286",

number.of.studies = T,

cex=1)

#forest plot code

forest(m.netmeta,

reference.group = "CT",

smlab = paste("treatment vs CT"),

drop.reference.group = TRUE,

label.left = "HR",

col.square = "#5C8286",

drop = TRUE,

sortvar = -lnHR,

label.right = "95% CrI")

#netleague display plot

pheatmap(data, cluster_rows = FALSE,cluster_cols =FALSE,treeheight_row = 15,

border =TRUE,legend = TRUE,

show_colnames=FALSE,show_rownames = FALSE,

col = colorRampPalette(colors = c("white","white","red"))(50),

fontsize_row=5,fontsize_col = 3,

fontsize_number = 8,display_numbers = txtlabel,

fontfamily= "serif",

xlab = "", ylab = "",

main = "")

#SUCRA plot

rank = netrank(m.netmeta,method = "SUCRA", small.values = "good",common = TRUE)

score = sort(rank$ranking.common,decreasing = TRUE)

dataframe = data.frame(score=as.numeric(score),name=factor(names(score),levels=names(score)))

ggplot(dataframe, aes(x=name, y = score)) +

geom_bar(stat='identity',width = 0.5,col="#5C8286") +

geom_text(aes(label=ifelse(round(score,3)==0,0.001,round(score,3))), vjust = -1,family = "serif")+

theme_classic()+

labs( y = 'rank probability (%)',x="") +

theme(axis.text.x = element_text(family = "Times New Roman",angle = 45, hjust = 1,size=8),

axis.text.y = element_text(family = "Times New Roman"),

axis.title = element_text(family = "Times New Roman"),

panel.grid.major = element_blank(),

panel.grid.minor = element_blank(),

legend.text = element_text(family = "Times New Roman",face = "italic"),

plot.title=element_text(family = "Times New Roman",hjust=0.5,color="black"),

axis.line = element_line(color="black",linewidth =1))
